# Long noncoding RNA *NNT-AS1* enhances the malignant phenotype of bladder cancer by acting as a competing endogenous RNA on microRNA-496 thereby increasing HMGB1 expression

**DOI:** 10.18632/aging.102591

**Published:** 2019-12-17

**Authors:** Deyao Wu, Tielong Zhang, Jie Wang, Jian Zhou, Huixing Pan, Ping Qu

**Affiliations:** 1 Department of Urology, The Fourth Affiliated Hospital of Nantong Medical College, Yancheng People’s Hospital, Yancheng 224001, China; 2 Department of Urology, Jianhu Hospital Affiliated to Nantong University, Yancheng 224700, China

**Keywords:** nicotinamide nucleotide transhydrogenase antisense RNA 1, bladder cancer, microRNA-496, high mobility group box 1, anticancer therapy

## Abstract

The long noncoding RNA nicotinamide nucleotide transhydrogenase antisense RNA 1 (*NNT-AS1*) is a key malignancy regulator in a variety of human cancers. In this study, we first measured the expression of *NNT-AS1* in bladder cancer and examined its role in cancer progression. The mechanisms behind the oncogenic functions of *NNT-AS1* in bladder cancer were explored. We found that *NNT-AS1* was upregulated in bladder cancer tissues and cell lines. This increased expression demonstrated a significant correlation with advanced clinical stage, lymph node metastasis, and shorter overall survival. *NNT-AS1* knockdown suppressed bladder cancer cell proliferation, migration, and invasion and facilitated apoptosis i*n vitro* and hindered tumor growth *in vivo*. *NNT-AS1* functioned as a competing endogenous RNA for microRNA-496 (miR-496), and the suppressive effects of *NNT-AS1* knockdown on malignant characteristics were abrogated by miR-496 silencing. *HMGB1* was identified as a direct target gene of miR-496 in bladder cancer, and HMGB1 expression was enhanced by *NNT-AS1* via sponging of miR-496. In conclusion, the *NNT-AS1*–miR-496–HMGB1 pathway plays a significant role in the aggressive behavior of bladder cancer and may lead to new *NNT-AS1*–based diagnostics and therapeutics.

## INTRODUCTION

Globally bladder cancer is the tenth most common malignant tumor in women and the fourth most common in men [1, 2]. The main histological type of bladder cancer is transitional cell carcinoma (TCC), which accounts for more than 90% of bladder cancer cases [3]. Many factors are involved in the carcinogenesis and progression of bladder cancer, including chromosomal aberrations, genetic polymer phisms, as well as genetic and epigenetic alterations [4]. Bladder cancer can be subdivided into three subtypes: superficial, invasive, and metastatic [5]. At diagnosis, 75% of cases are classified as superficial tumors, 20% are invasive cancers, and 5% already involve metastasis [6, 7]. The current gold-standard treatment of bladder cancer is a surgical operation followed by radio-chemotherapy and biological therapy [8]. Surgical resection is the key modality, and adjuvant therapy is considered an effective supplementary treatment for preventing recurrence and metastasis [9]. Nonetheless, more than 30% of patients either fail to respond to treatment or experience recurrent disease within five years, and 50% of patients die of metastatic disease [10]. Improving our understanding of the molecular mechanisms underlying the carcinogenesis and progression of bladder cancer may help identify new therapeutic strategies against bladder cancer.

Long noncoding RNAs (lncRNAs) have recently emerged as a novel hot area of research into anticancer therapies [11]. LncRNAs are a family of RNA transcripts that are > 200 nucleotides long [12]. They lack protein-coding ability yet participate in the control of a variety of cellular processes, including epigenetic, transcriptional, and post-transcriptional regulation [13]. Increasing evidence has shown involvement of lncRNAs in the malignant characteristics of human cancers, including bladder cancer [14–16]. Numerous lncRNAs are differentially expressed in bladder cancer, including *PART1* [17], *HCG22* [18], and *TUC338* [19]. The dysregulated lncRNAs exert crucial effects on bladder carcinogenesis and cancer progression through various mechanisms [20–22]. They can function as guides, scaffolds, and molecular sponges in interactions with proteins, microRNAs (miRNAs), and mRNAs, thereby resulting in the formation of a complex signal-regulating network [23, 24].

MiRNAs belong to a large group of single-stranded noncoding short RNAs 17–24 nucleotides in length [25]. MiRNAs directly interact with the 3′-untranslated region (3′-UTR) of their target mRNAs, thus degrading these mRNAs and/or inhibiting translation [26]. Studies have revealed changes in miRNA expression in bladder cancer, suggesting that miRNAs take part in the initiation and progression of this disease [27–29]. MiRNAs can exert tumor-suppressive or oncogenic actions in bladder cancer and participate in the modulation of a wide range of pathological conditions [30–32]. Therefore, clarifying the associations among lncRNAs, miRNAs, and bladder cancer may facilitate the development of novel techniques for the prevention, diagnosis, and treatment of this condition.

An lncRNA called nicotinamide nucleotide transhydrogenase antisense RNA 1 (*NNT-AS1*) is abnormally expressed in a variety of human cancers and functions as a key regulator of cancer progression [33–42]. Nevertheless, the expression profile, clinical significance, and biological functions of *NNT-AS1* in bladder cancer and the underlying mechanisms remain unknown. In the present study, we first measured the expression of *NNT-AS1* in bladder cancer tissues and cell lines. Next, we examined the clinical value of *NNT-AS1* among patients with bladder cancer. Furthermore, the specific roles and mechanisms underlying the oncogenic activities of *NNT-AS1* in bladder cancer were explored in detail.

## RESULTS

### *NNT-AS1* is overexpressed in bladder cancer

**Figure 1:**
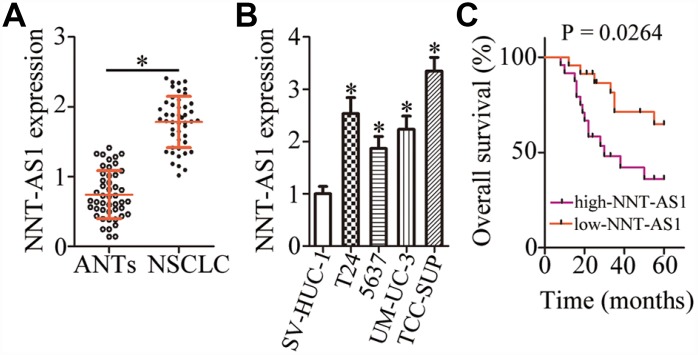
***NNT-AS1* is overexpressed in bladder cancer and is associated with poor clinical outcomes.** (**A**) The expression of *NNT-AS1* in the 47 pairs of bladder cancer tissue specimens and matched adjacent normal tissues (ANTs) was determined by RT-qPCR. *P < 0.05 vs. the ANTs group. (**B**) *NNT-AS1* levels were measured in four bladder cancer cell lines and a normal bladder immortalized epithelial cell line (SV-HUC-1) by RT-qPCR. *P < 0.05 vs. group SV-HUC-1. (**C**) Kaplan–Meier plot demonstrating the association between *NNT-AS1* expression and overall survival of the patients with bladder cancer. P = 0.0264.

To determine the expression profile of *NNT-AS1* in bladder cancer, we measured its expression in 47 pairs of bladder cancer tissue specimens and matched adjacent normal tissues (ANTs) by reverse-transcription quantitative PCR (RT-qPCR). *NNT-AS1* was found to be overexpressed in bladder cancer tissues relative to ANTs (Figure 1A, P < 0.05). In addition, obviously higher expression of *NNT-AS1* was detected in all four bladder cancer cell lines (T24, 5637, UM-UC-3, and TCC-SUP) when compared with a normal bladder immortalized epithelial cell line (SV-HUC-1; Figure 1B, P < 0.05).

We next determined the clinical significance of *NNT-AS1* in patients with bladder cancer. All these patients (n = 47) were distributed into two groups: either “high-NNT-AS1” (n = 24) or “low-NNT-AS1” (n = 23), based on the median value of *NNT-AS1* expression in the bladder cancer tissue specimens. Evaluation of the correlation between *NNT-AS1* expression and clinical parameters revealed that high *NNT-AS1* expression significantly correlated with lymphatic invasion (P = 0.017) and TNM stage (P = 0.015) in patients with bladder cancer (Table 1). Furthermore, patients in the high-NNT-AS1 group demonstrated shorter overall survival in comparison with the patients in the low-NNT-AS1 group (Figure 1C, P = 0.0264). Taken together, these results indicated that *NNT-AS1* was overexpressed in bladder cancer and correlated with poor clinical outcomes, suggesting that this lncRNA may be closely related to the malignancy of bladder cancer.

**Table 1:** Correlation between *NNT-AS1* expression and clinical parameters of patients with bladder cancer.

**Clinical parameters**	***NNT-AS1* expression**		**P**
	**High**	**Low**	
**Age (years)**			0.461
< 60	18 (75.0%)	20 (87.0%)	
≥ 60	6 (25.0%)	3 (13.0%)	
Gender			0.534
Male	15 (62.5%)	17 (73.9%)	
Female	9 (37.5%)	6 (26.1%)	
**Histologic grade**			0.212
Low grade	10 (41.7%)	5 (21.7%)	
High grade	14 (58.3%)	18 (78.3%)	
**Lymphatic invasion**			0.017
Negative	14 (58.3%)	21 (91.3%)	
Positive	10 (41.7%)	2 (8.7%)	
**TNM stage**			0.015
I-II	11 (45.8%)	19 (82.6%)	
III-IV	13 (54.2%)	4 (17.4%)	
**Smoking**			0.380
Nonsmoking	12 (50.0%)	15 (65.2%)	
Smoking	12 (50.0%)	8 (34.8%)	

### Knockdown of *NNT-AS1* restricts bladder cancer cell proliferation, migration, and invasion but induces apoptosis

**Figure 2:**
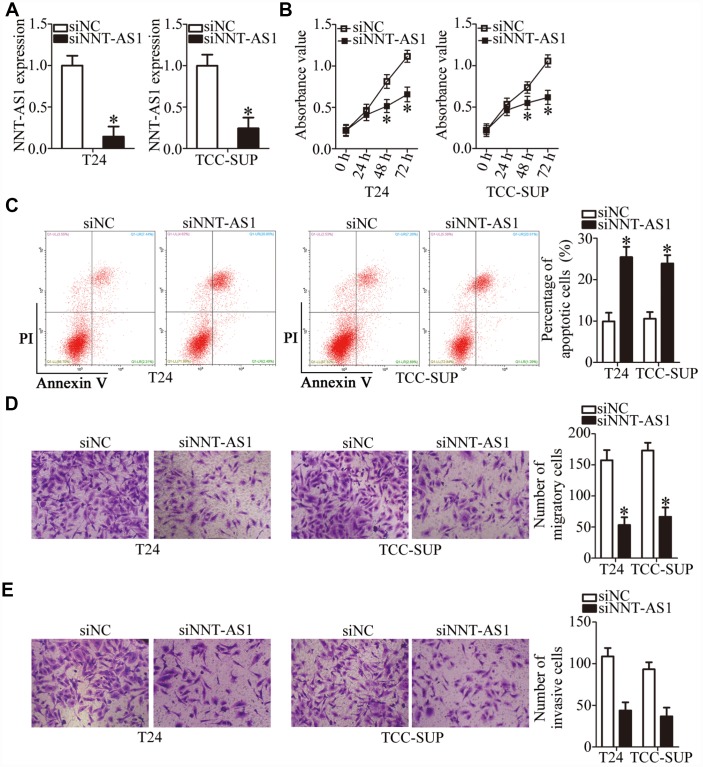
**Downregulation of *NNT-AS1* inhibits the malignant characteristics of bladder cancer cells *in vitro.*** (**A**) RT-qPCR was carried out to determine the expression of *NNT-AS1* in T24 and TCC-SUP cells after either siNNT-AS1 or siNC transfection. *P < 0.05 vs. group siNC. (**B**, **C**) The proliferation and apoptosis status of *NNT-AS1*–depleted T24 and TCC-SUP cells were tested via the CCK-8 assay and flow cytometry. *P < 0.05 vs. group siNC. (**D**, **E**) The migration and invasion abilities of T24 and TCC-SUP cells after *NNT-AS1* knockdown were evaluated using transwell cell migration and invasion assays. *P < 0.05 vs. the siNC group.

T24 and TCC-SUP showed the highest expression of *NNT-AS1* among the four bladder cancer cell lines; accordingly, they were chosen for further experiments. To investigate whether *NNT-AS1* is functionally implicated in the aggressiveness of bladder cancer, either small interfering RNAs (siRNAs) specific to *NNT-AS1* (siNNT-AS1) or a negative control (NC) siRNA (siNC) were transfected into T24 and TCC-SUP cells, and the efficiency of siRNA transfection was assessed by RT-qPCR. The siNNT-AS1 transfection dramatically reduced the expression of *NNT-AS1* in both T24 and TCC-SUP (Figure 2A, P < 0.05). The impact of *NNT-AS1* knockdown on the proliferation of bladder cancer cells was determined using a Cell Counting Kit-8 (CCK-8) assay. *NNT-AS1* knockdown significantly hindered the proliferation of T24 and TCC-SUP cells compared with the siNC group (Figure 2B, P < 0.05). Next, flow cytometry was utilized to test whether the influence of *NNT-AS1* on bladder cancer cell proliferation is related to apoptosis. Transfection with siNNT-AS1 clearly raised the proportion of apoptotic T24 and TCC-SUP cells (Figure 2C, P < 0.05), indicating that the inhibition of bladder cancer cell proliferation by *NNT-AS1* knockdown could be attributed to the promotion of apoptosis. Using transwell cell migration and invasion assays, we evaluated the migratory (Figure 2D, P < 0.05) and invasive (Figure 2E, P < 0.05) abilities of *NNT-AS1*–depleted T24 and TCC-SUP cells. Upon knockdown of *NNT-AS1*, the migration and invasiveness of T24 and TCC-SUP cells significantly decreased as compared with the siNC group. Overall, these data suggested that *NNT-AS1* may promote bladder cancer progression.

### *NNT-AS1* acts as a competing endogenous RNA (ceRNA) on miR-496 in bladder cancer cells

**Figure 3:**
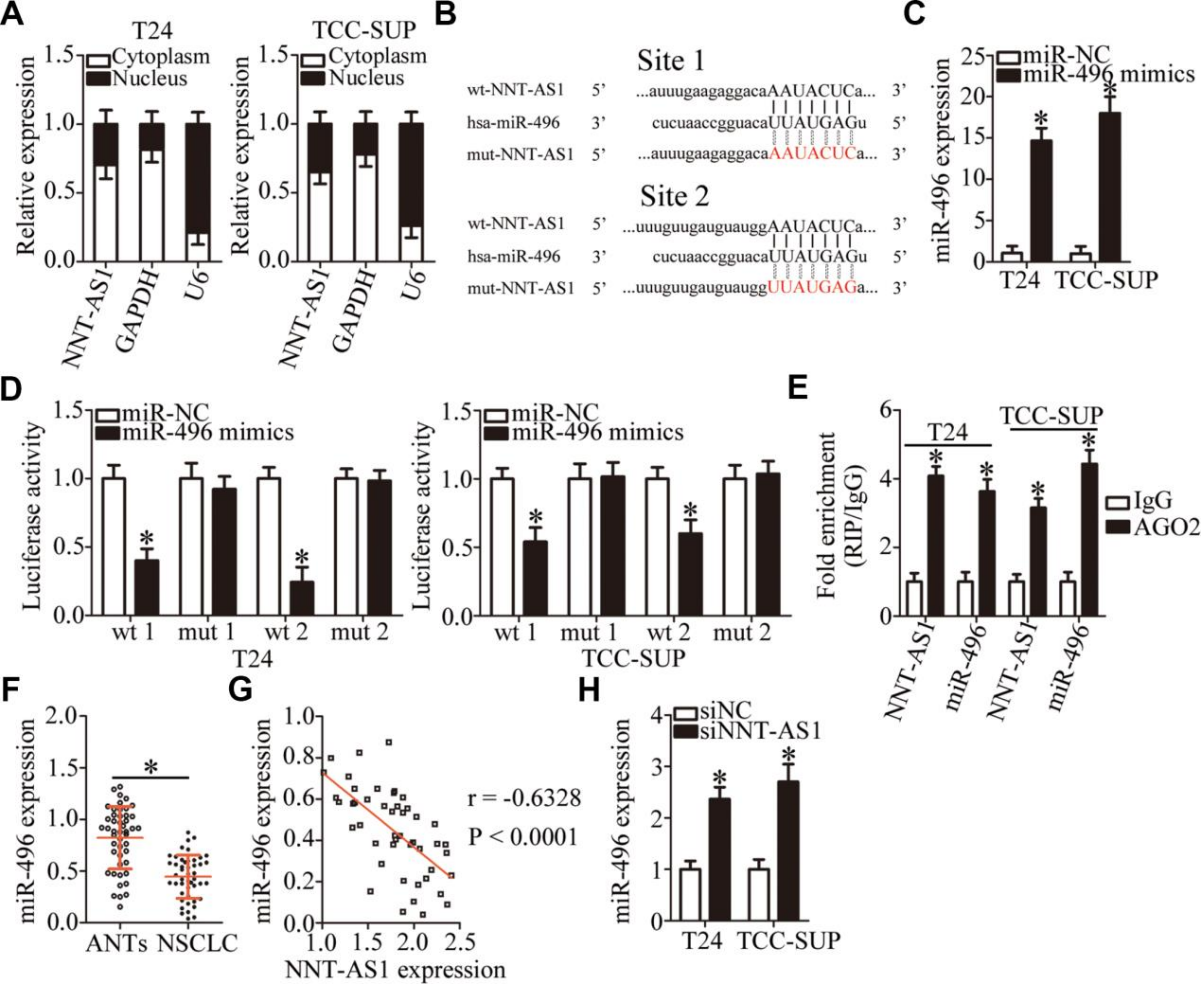
***NNT-AS1* serves as a competing endogenous RNA (ceRNA) for miR-496 in bladder cancer cells.** (**A**) Relative *NNT-AS1* expression in nuclear and cytoplasmic fractions of T24 and TCC-SUP cells. (**B**) Bioinformatics prediction via starBase 3.0 uncovered two possible binding sites for miR-496 in *NNT-AS1*. (**C**) RT-qPCR was conducted to analyze miR-496 expression in T24 and TCC-SUP cells after introduction of either the miR-496 mimics or miR-NC. *P < 0.05 vs. group miR-NC. (**D**) Either plasmid wt-NNT-AS1 or mut-NNT-AS1 was cotransfected into T24 and TCC-SUP cells with either the miR-496 mimics or miR-NC for the measurement of luciferase activity. *P < 0.05 vs. the miR-NC group. (**E**) A RIP assay was carried out to determine the interaction between miR-496 and *NNT-AS1* in T24 and TCC-SUP cells. *P < 0.05 vs. group IgG. (**F**) MiR-496 expression in 47 pairs of bladder cancer tissues and ANTs was assessed via RT-qPCR. *P < 0.05 vs. group ANTs. (**G**) The correlation between miR-496 and *NNT-AS1* expression levels in the 47 bladder cancer tissue specimens was examined by Spearman’s correlation analysis. r = -0.6328, P < 0.0001. (**H**) The expression of miR-496 in *NNT-AS1*–depleted T24 and TCC-SUP cells was quantified by RT-qPCR. *P < 0.05 vs. siNC.

To investigate the mechanisms by which *NNT-AS1* promotes the malignant behaviors of bladder cancer cells, we first analyzed the subcellular localization of *NNT-AS1* in these cells. We found that *NNT-AS1* is mainly located in the cytoplasm of T24 and TCC-SUP cells (Figure 3A). Growing evidence suggests that cytoplasmic lncRNAs can function as ceRNAs by competitively interacting with specific miRNAs [43–45]. A bioinformatic algorithm, starBase 3.0, was executed to search for a potential miRNA target of *NNT-AS1*. MiR-496 (Figure 3B) was predicted to contain (with high probability) a binding site for *NNT-AS1* and was selected for validation as it has been reported to participate in tumorigenesis and tumor progression [46–49]. A luciferase reporter assay was performed on bladder cancer cells to test the miR-496–binding site in *NNT-AS1*. Either miR-496 mimics or miR-NC and either plasmid wt-NNT-AS1 (containing wild-type miR-496–binding site 1 or site 2; Figure 3B) or plasmid mut-NNT-AS1 (containing mutant miR-496–binding site 1 or site 2; Figure 3B) were cotransfected into T24 and TCC-SUP cells. The miR-496 mimics’ dramatic transfection–mediated overexpression of miR-496 (Figure 3C, P < 0.05) decreased the luciferase activity generated by the reporter plasmid wt-NNT-AS1 (carrying site 1 or site 2) in T24 and TCC-SUP cells (P < 0.05; Figure 3D). By contrast, miR-496 upregulation did not reduce the luciferase activity generated by mut-NNT-AS1 (carrying site 1 or site 2; Figure 3D). In addition, an RNA immunoprecipitation (RIP) assay was carried out to determine the interaction between miR-496 and *NNT-AS1* in bladder cancer cells. MiR-496 and *NNT-AS1* were specifically enriched in an Argonaute 2 (AGO2) immunoprecipitate from the lysates of T24 and TCC-SUP cells as compared with the IgG control group (Figure 3E, P < 0.05).

MiR-496 expression was then quantified in the 47 pairs of bladder cancer tissue specimens and matched ANTs. The results of RT-qPCR analysis revealed that miR-496 expression was significantly lower in bladder cancer tissues compared with that in ANTs (Figure 3F, P < 0.05) was inversely correlated with *NNT-AS1* expression (Figure 3G; r = -0.6328, P < 0.0001). Finally, RT-qPCR analysis was performed to determine whether *NNT-AS1* can sponge miR-496 in bladder cancer cells. The expression of miR-496 was substantially higher in the *NNT-AS1*–depleted T24 and TCC-SUP cells (Figure 3H, P < 0.05). Altogether, these results suggested that *NNT-AS1* acts as a ceRNA and sponges miR-496 in bladder cancer cells.

### *HMGB1* is a direct target gene of miR-496 in bladder cancer cells

**Figure 4:**
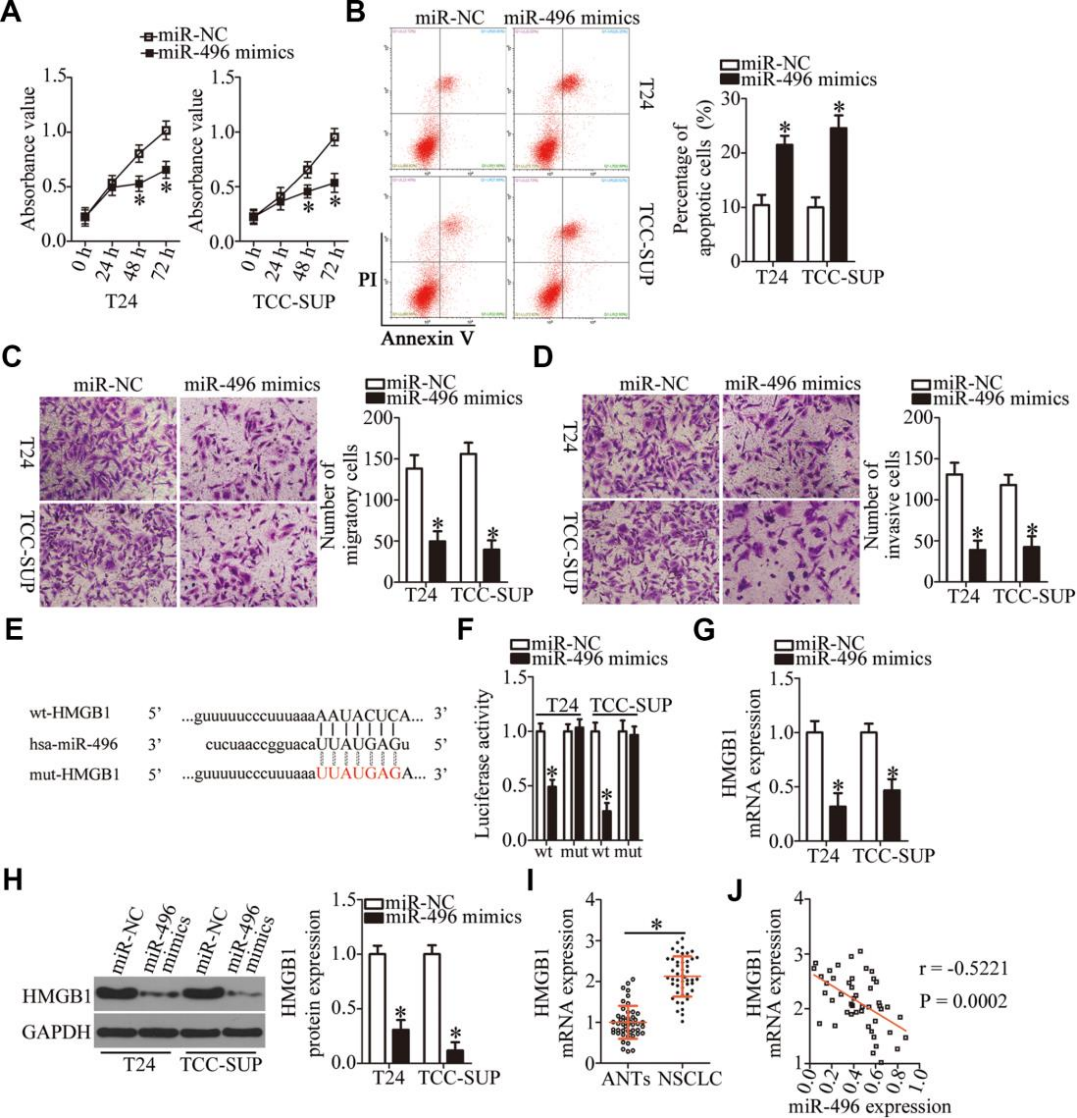
***HMGB1* mRNA is a direct target of miR-496 in bladder cancer cells.** (**A**) CCK-8 assay of the proliferation of T24 and TCC-SUP cells transfected with either the miR-496 mimics or miR-NC. *P < 0.05 vs. miR-NC. (**B**) The proportion of apoptotic miR-496–overexpressing T24 and TCC-SUP cells was detected by flow-cytometric analysis. *P < 0.05 vs. group miR-NC. (**C**, **D**) The migratory and invasive abilities were examined in transwell migration and invasion assays involving T24 and TCC-SUP cells transfected with either the miR-496 mimics or miR-NC. *P < 0.05 vs. the miR-NC group. (**E**) The predicted wild-type and mutant miR-496–binding sequences in the 3′-UTR of *HMGB1*. (**F**) A reporter plasmid containing either a wild-type or mutant *HMGB1* 3′-UTR fragment was cotransfected in combination with either the miR-496 mimics or miR-NC into T24 and TCC-SUP cells, and luciferase activity was quantified. *P < 0.05 vs. the miR-NC group. (**G**, **H**) Detection of *HMGB1* mRNA and protein expression levels in miR-496–overexpressing T24 and TCC-SUP cells by RT-qPCR and western blot analysis, respectively. *P < 0.05 vs. group miR-NC. (**I**) *HMGB1* mRNA expression was analyzed by RT-qPCR in the 47 pairs of bladder cancer tissue specimens and ANTs. *P < 0.05 vs. group ANTs. (**J**) Assessment of the correlation between miR-496 expression and *HMGB1* mRNA expression among the 47 bladder cancer tissue specimens was performed via Spearman’s correlation analysis. r = -0.5221, P = 0.0002.

We then explored the biological functions of miR-496 in bladder cancer cells. T24 and TCC-SUP cells were transfected with either the miR-496 mimics or miR-NC, and then a series of functional experiments were carried out with the transfected cells. The CCK-8 assay and flow cytometry revealed that miR-496 upregulation significantly decreased proliferation (Figure 4A, P < 0.05) and increased apoptosis (Figure 4B, P < 0.05) of T24 and TCC-SUP cells. Additionally, ectopic miR-496 expression markedly decreased the number of migratory and invasive T24 and TCC-SUP cells, suggesting that miR-496 impaired the migration (Figure 4C, P < 0.05) and invasiveness (Figure 4D, P < 0.05) of bladder cancer cells. These findings suggested that miR-496 performs tumor-suppressive activities in bladder cancer cells by inhibiting cell proliferation, migration, and invasion and by promoting apoptosis.

To elucidate the mechanisms behind miR-496–mediated inhibition of bladder cancer progression, three miRNA target prediction databases were searched to predict functionally relevant targets of miR-496. The bioinformatics prediction showed that the 3′-UTR of *HMGB1* mRNA matches the “seed sequence” of miR-496 (Figure 4E). To evaluate the possibility of binding between miR-496 and the 3′-UTR of *HMGB1* mRNA, a luciferase reporter assay was performed on T24 and TCC-SUP cells after cotransfection with either the miR-496 mimics or miR-NC and either plasmid wt-HMGB1 (expressing luciferase mRNA containing the wild-type miR-496–binding site in the 3′-UTR of *HMGB1*) or the plasmid mut-HMGB1 (expressing luciferase mRNA containing a mutant miR-496–binding site in the 3′-UTR of *HMGB1*). The luciferase activity generated by the wt-HMGB1 reporter plasmid in T24 and TCC-SUP cells was strikingly decreased by miR-496 upregulation (P < 0.05), whereas the luciferase activity generated by the plasmid mut-HMGB1 was unaltered by cotransfection of miR-496 mimics (Figure 4F). In addition, resumption of miR-496 expression notably decreased HMGB1 expression at both mRNA (Figure 4G, P < 0.05) and protein (Figure 4H, P < 0.05) levels in T24 and TCC-SUP cells, as evidenced by RT-qPCR and western blotting. Furthermore, we found that *HMGB1* mRNA was more strongly expressed in bladder cancer tissues than in ANTs (Figure 4I, P < 0.05). The high expression of HMGB1 showed a negative correlation with miR-496 expression among the 47 bladder cancer tissue specimens (Figure 4J; r = -0.5221, P = 0.0002). Collectively, these data suggested that *HMGB1* mRNA is a direct target of miR-496 in bladder cancer cells.

### Tumor-suppressive effects of miR-496 in bladder cancer cells are mediated by downregulation of HMGB1

**Figure 5:**
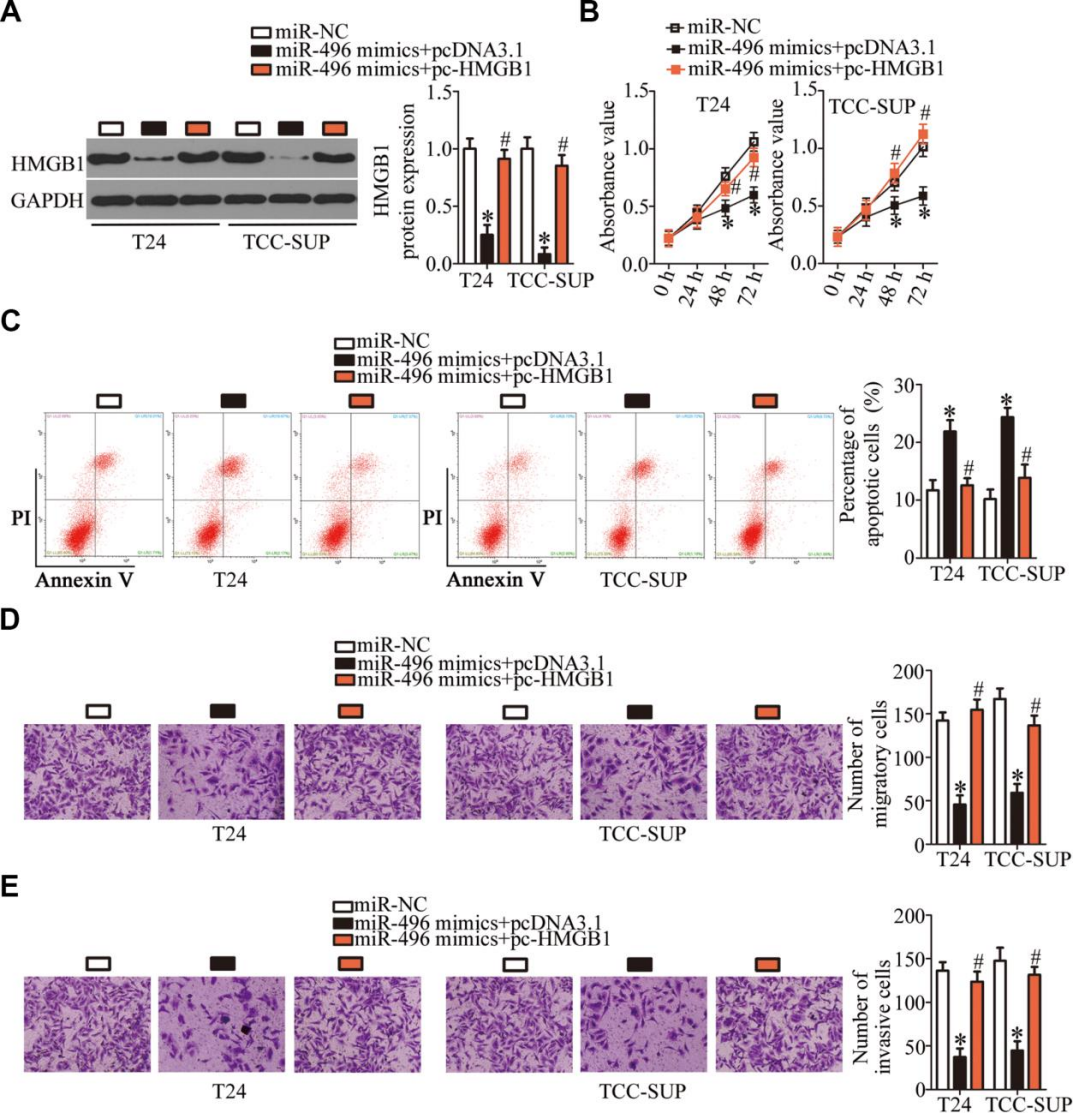
**MiR-496 performs its tumor-suppressive actions in bladder cancer cells by decreasing HMGB1 expression.** (**A**) The miR-496 mimics in combination with either the HMGB1-overexpressing plasmid pc-HMGB1 or the empty pcDNA3.1 vector was cotransfected into T24 and TCC-SUP cells. At 72 h post-transfection, western blotting was performed to analyze HMGB1 expression. *P < 0.05 vs. the miR-NC group. ^#^P < 0.05 vs. the miR-496 mimics+pcDNA3.1 group. (**B**, **C**) The proliferative and apoptotic activities of T24 and TCC-SUP cells after cotransfection with the miR-496 mimics and either pc-HMGB1 or pcDNA3.1 were evaluated through the CCK-8 assay and flow-cytometric analysis, respectively. *P < 0.05 vs. the miR-NC group. ^#^P < 0.05 vs. group miR-496 mimics+pcDNA3.1. (**D**, **E**) Transwell migration and invasion assays were conducted to examine the migratory and invasive abilities of T24 and TCC-SUP cells after cotransfection with the miR-496 mimics and either pc-HMGB1 or pcDNA3.1. *P < 0.05 vs. group miR-NC. ^#^P < 0.05 vs. the miR-496 mimics+pcDNA3.1 group.

To investigate whether the miR-496–driven suppression of bladder cancer progression was mediated via direct targeting of *HMGB1* mRNA, rescue experiments were conducted with T24 and TCC-SUP cells cotransfected with the miR-496 mimics and either the HMGB1-overexpressing plasmid pcDNA3.1-HMGB1 (hereafter: pc-HMGB1) or the empty pcDNA3.1 vector. The decrease in HMGB1 expression caused by miR-496 overexpression was reversed in T24 and TCC-SUP cells after cotransfection with pc-HMGB1 (Figure 5A, P < 0.05), as revealed by western blotting. Furthermore, functional experiments showed that miR-496 overexpression attenuated T24 and TCC-SUP cell proliferation (Figure 5B, P < 0.05), promoted apoptosis (Figure 5C, P < 0.05), and reduced cell migration (Figure 5D, P < 0.05) and invasion (Figure 5E, P < 0.05). These phenomena were abrogated by reintroduction of HMGB1 expression. Thus, miR-496 was confirmed as a tumor-suppressive miRNA inhibiting the malignant characteristics of bladder cancer cells at least partly by decreasing HMGB1 expression.

### A reduction in *NNT-AS1* expression suppresses the malignant phenotype of bladder cancer cells by inhibiting miR-496–HMGB1 axis output

**Figure 6:**
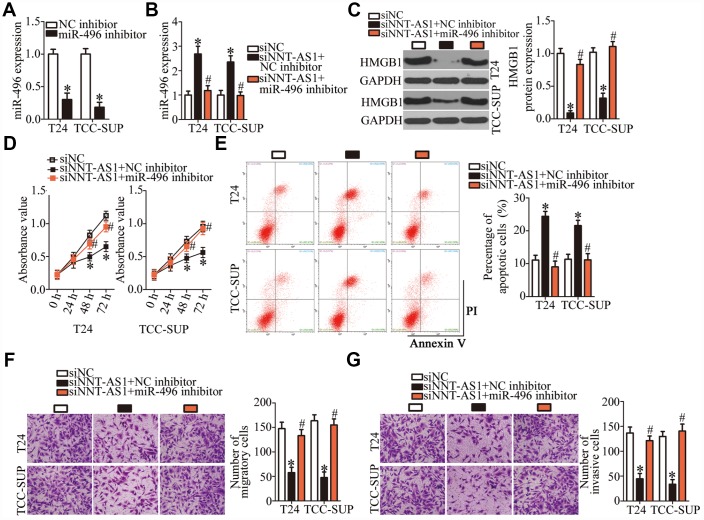
**The oncogenic functions of *NNT-AS1* in bladder cancer cells are mediated by stimulation of the miR-496–HMGB1 axis output.** (**A**) T24 and TCC-SUP cells were transfected with either the miR-496 inhibitor or NC inhibitor. After 48 h, the transfection efficiency was assessed by RT-qPCR. *P < 0.05 vs. NC inhibitor. (**B**, **C**) siNNT-AS1 plus either the miR-496 inhibitor or NC inhibitor were cotransfected into T24 and TCC-SUP cells. The miR-496 and HMGB1 protein levels were measured by RT-qPCR and western blotting, respectively. *P < 0.05 vs. group siNC. ^#^P < 0.05 vs. group siNNT-AS1+NC inhibitor. (**D**–**G**) CCK-8 assay, flow-cytometric analysis, and transwell migration and invasion assays were performed to determine the status of proliferation, apoptosis, migration, and invasiveness of T24 and TCC-SUP cells that were cotransfected with siNNT-AS1 and either the miR-496 inhibitor or NC inhibitor. *P < 0.05 vs. the siNC group. ^#^P < 0.05 vs. group siNNT-AS1+NC inhibitor.

A series of rescue experiments were conducted to determine whether the oncogenic roles of *NNT-AS1* in bladder cancer progression were mediated by the miR-496–HMGB1 pathway. First, the transfection efficiency of the miR-496 inhibitor was assessed by RT-qPCR (Figure 6A, P < 0.05). After that, siNNT-AS1 in combination with either the miR-496 inhibitor or NC inhibitor was introduced into T24 and TCC-SUP cells. As shown by RT-qPCR, miR-496 inhibitor cotransfection reversed the stimulatory effect of *NNT-AS1* knockdown on miR-496 expression in T24 and TCC-SUP cells (Figure 6B, P < 0.05). Similarly, the decrease in HMGB1 protein expression caused by siNNT-AS1 was reversed in T24 and TCC-SUP cells after cotransfection with the miR-496 inhibitor (Figure 6C, P < 0.05). Furthermore, the effects of *NNT-AS1* knockdown on the proliferation (Figure 6D, P < 0.05), apoptosis (Figure 6E, P < 0.05), migration (Figure 6F, P < 0.05), and invasiveness (Figure 6G, P < 0.05) of T24 and TCC-SUP cells were neutralized by the miR-496 inhibitor. Thus, these findings revealed that the miR-496–HMGB1 axis was essential for the effects of *NNT-AS1* on the malignant characteristics of bladder cancer cells.

### *NNT-AS1* knockdown decreases the tumor growth of bladder cancer cells *in vivo*

**Figure 7:**
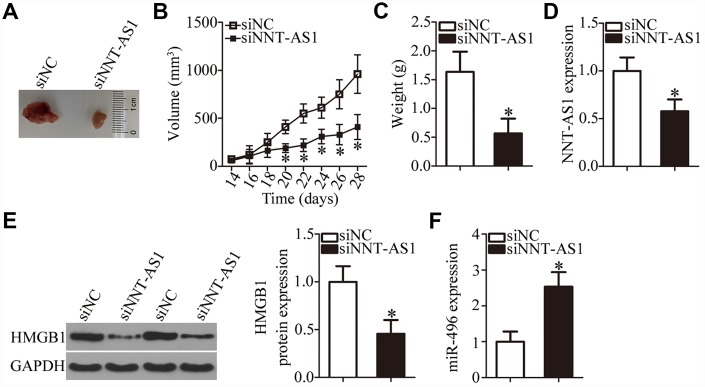
**Knockdown of *NNT-AS1* impairs bladder cancer cell growth *in vivo*.** All the mice were injected with T24 cells that were transfected with either siNNT-AS1 or siNC. (**A**) Representative images of tumor xenografts collected from groups siNNT-AS1 and siNC. (**B**) The growth curves of subcutaneous tumor xenografts in groups siNNT-AS1 and siNC. *P < 0.05 vs. the siNC group. (**C**) The weights of tumor xenografts derived from either siNNT-AS1–transfected or siNC-transfected T24 cells were measured at 4 weeks post-inoculation. *P < 0.05 vs. group siNC. (**D**) *NNT-AS1* expression in tumor xenografts obtained from groups siNNT-AS1 and siNC was evaluated via RT-qPCR. *P < 0.05 vs. the siNC group. (**E**) Western-blotting assessment of HMGB1 expression in the tumor xenografts derived from either siNNT-AS1–transfected or siNC-transfected T24 cells. *P < 0.05 vs. the siNC group. (**F**) MiR-496 expression in tumor xenografts obtained from both groups was evaluated via RT-qPCR. *P < 0.05 vs. the siNC group.

A tumor xenograft assay was carried out to test the influence of *NNT-AS1* on the tumor growth of bladder cancer cells *in vivo*. All nude mice were randomly subdivided into two groups: One group was inoculated with siNNT-AS1–transfected T24 cells; T24 cells transfected with siNC were injected into the mice in the other group. On day 28, all the mice were euthanized, and the tumor xenografts were resected and weighed. Representative images are presented in Figure 7A. The volume of tumor xenografts in the siNNT-AS1 group was notably smaller in comparison with the siNC group (Figure 7B, P < 0.05). The weight of the tumor xenografts was significantly lower in the siNNT-AS1 group compared with the siNC group (Figure 7C, P < 0.05). Total RNA and protein were then extracted from tumor xenografts, and *NNT-AS1*, miR-496, and HMGB1 protein expression were measured. This analysis indicated that tumor xenografts derived from siNNT-AS1–transfected T24 cells featured lower *NNT-AS1* (Figure 7D, P < 0.05) and HMGB1 protein expression (Figure 7E, P < 0.05) as well as higher miR-496 expression (Figure 7F, P < 0.05) compared with the siNC group. Overall, these data indicated that the *NNT-AS1* knockdown restricted the growth of bladder cancer cells *in vivo* through the miR-496–HMGB1 axis.

## DISCUSSION

LncRNAs have been attracting increasing attention in recent years [50, 51]. Abnormal expression of lncRNAs has been discovered in bladder cancer, and their aberrant functions play a key part in the genesis and progression of this cancer, with lncRNAs functioning as tumor suppressors or oncogenic RNA [52, 53]. Hence, investigation of the activities of lncRNAs in bladder cancer may help to identify effective targets for anticancer therapies. Nevertheless, only a handful of lncRNAs have been studied in detail. In this work, we first tested whether *NNT-AS1* is dysregulated in bladder cancer and assessed its clinical value among patients with bladder cancer. Second, we applied siRNA to silence endogenous *NNT-AS1* expression in bladder cancer cells in order to investigate the biological effects of *NNT-AS1* on the aggressive characteristics of these cells *in vitro* and *in vivo*. Third, the mechanisms underlying the activities of *NNT-AS1* in bladder cancer cells were explored.

*NNT-AS1* is upregulated in osteosarcoma, and this upregulation significantly correlates with tumor size, Enneking stage, and tumor metastasis [33, 34]. Remarkably, *NNT-AS1* is known as an independent and significant risk factor predicting survival among patients with osteosarcoma [33]. *NNT-AS1* is also overexpressed in gastric cancer [35, 36]. Increased expression of *NNT-AS1* is closely associated with the tumor stage, lymph node metastasis, and TNM stage [35]. Patients with gastric cancer featuring high *NNT-AS1* expression manifest shorter overall survival than do the patients with low *NNT-AS1* expression [35]. *NNT-AS1* is also highly expressed in non–small cell lung cancer [37, 38], ovarian cancer [39], breast cancer [40], hepatocellular carcinoma [41], and cervical cancer [42]. Nevertheless, whether *NNT-AS1* is dysregulated in bladder cancer has remained unknown. Our results indicated that the expression of *NNT-AS1* is high in bladder cancer tissues and cell lines. The high *NNT-AS1* expression correlated with the clinical stage and lymph node metastasis among our bladder cancer patients. Patients with bladder cancer in the high-NNT-AS1 group had a worse prognosis than those in the low-*NNT-AS1* group. Our findings suggest that *NNT-AS1* may be a promising biomarker for the diagnosis and prognosis of bladder cancer.

*NNT-AS1* performs an oncogenic function in osteosarcoma by promoting cell proliferation, migration, and invasion and by suppressing cell cycle arrest and apoptosis [33, 34]. *NNT-AS1* knockdown attenuates gastric cancer cell proliferation and invasion *in vitro*, promotes cell cycle arrest, and hinders *in vivo* tumor growth [35, 36]. In non–small cell lung cancer, knockdown of *NNT-AS1* inhibits cell proliferation, colony formation, and invasion and induces apoptosis, cell cycle arrest, and cisplatin chemoresistance [37, 38]. Nevertheless, the influence of *NNT-AS1* on the biological functions of bladder cancer has been unclear. In this study, a series of functional experiments revealed that *NNT-AS1* knockdown restricts cell proliferation, migration, and invasion and facilitates apoptosis *in vitro* as well as slows tumor growth *in vivo*.

One of the main roles of lncRNAs is functioning as a ceRNA in a regulatory network involving lncRNA, miRNA, and target mRNA [54]. Here, the underlying mechanisms of *NNT-AS1* activity in the malignancy of bladder cancer cells were explored. We demonstrated that *NNT-AS1* can raise HMGB1 expression by functioning as a ceRNA for miR-496. The latter is known to be downregulated in colorectal cancer [46], non–small cell lung cancer [47], glioma [48], and osteosarcoma [49]. MiR-496 exerts tumor-suppressive actions on these human cancer types [46–49]. In our study, for the first time, miR-496 was found to be downregulated in bladder cancer and to directly target *HMGB1* mRNA to restrain the aggressive phenotype of bladder cancer. Moreover, we found that miR-496 can be sponged by *NNT-AS1*, and the miR-496–HMGB1 axis is responsible for the oncogenic roles of *NNT-AS1*.

HMGB1, encoded within chromosomal region 8q22, is a highly conserved DNA-binding protein. It can relocate from the cytoplasm to the nucleus and interact with transcription factors, nucleosomes, and histones [55]. HMGB1 is upregulated in bladder cancer and is closely associated with the tumor grade and tumor stage [56, 57]. Patients with bladder cancer featuring high HMGB1 expression have shorter disease-free survival and overall survival [56]. In addition, HMGB1 has been confirmed as an independent prognostic factor in bladder cancer [56]. HMGB1 exerts oncogenic effects on the formation and progression of bladder cancer and participates in the modulation of tumor cell proliferation, apoptosis, cell cycle, metastasis, radioresistance, and tumorigenesis [58]. In this study, we illustrated a novel upstream mechanism regulating the expression of HMGB1 in bladder cancer cells *in vitro* and *in vivo*. *NNT-AS1*, which contains an miR-496–binding site, was found to function as a ceRNA and to sponge miR-496, thereby increasing HMGB1 expression.

A weakness of the manuscript was that the bladder cancer samples have been studied from the same discovery cohort of patients as those assessed for survival and clinical behavior. A separate cohort of patients independent of the discovery cohort would have been studied as a validation cohort. We will resolve this weakness in our further investigations.

In summary, our results revealed that knockdown of *NNT-AS1* suppresses the malignant phenotype of bladder cancer cells *in vitro* and *in vivo*. In terms of the mechanism, *NNT-AS1* acts as a ceRNA on miR-496, thereby reversing the tumor-suppressive influence of miR-496 on HMGB1 expression. Thus, the *NNT-AS1*–miR-496–HMGB1 pathway is an important player in the malignancy of bladder cancer, suggesting that this pathway may be an effective target for anticancer therapies.

## MATERIALS AND METHODS

### Ethics statement

This investigation was conducted in accordance with the ethical standards of the Declaration of Helsinki and national and international guidelines and was approved by the authors' institutional review board. This study was approved by the Ethics Committee of the Fourth Affiliated Hospital of Nantong Medical College. Informed consent was obtained. The experimental procedures involving animals were approved by the Animal Ethics Committee of the Fourth Affiliated Hospital of Nantong Medical College. Every effort was made to minimize the suffering of the mice.

### Clinical specimens

Sample were collected from consenting patients. Forty-seven pairs of bladder cancer tissue specimens and matched ANTs were obtained from the hospital between May 2013 to June 2014. Patients who were treated with preoperative radiotherapy, chemotherapy, or other anticancer modalities were excluded from this study. All tumor specimens were immediately frozen and stored in liquid nitrogen until RNA isolation.

### Cell culture

Four human bladder cancer cell lines (T24, 5637, UM-UC-3, and TCC-SUP) and a normal bladder immortalized epithelial cell line (SV-HUC-1) were used in this study. These cell lines were bought from the Shanghai Institute of Biochemistry and Cell Biology (Shanghai, China) and were maintained in Dulbecco’s modified Eagle’s medium (DMEM) containing 10% of fetal bovine serum (FBS), 100 U/ml penicillin, and 100 μg/ml streptomycin (all from Gibco; Thermo Fisher Scientific, Inc., Waltham, MA, USA). All the cells were kept in a humidified incubator with 5% circulating CO_2_ at 37°C.

### Transient transfection

The siRNAs specific to *NNT-AS1* (siNNT-AS1) and negative control (NC) siRNA (siNC) were purchased from Guangzhou Ribobio Technology (Guangzhou, China). The miR-496 mimics, NC miRNA mimics (miR-NC), miR-496 inhibitor, and NC inhibitor were chemically synthesized by Shanghai GenePharma Technology (Shanghai, China). The HMGB1-overexpressing plasmid (pc-HMGB1) and the empty pcDNA3.1 vector were acquired from Shanghai Sangon Biotech Co., Ltd. (Shanghai, China). All transient transfection procedures were carried out using Lipofectamine® 2000 (Invitrogen; Thermo Fisher Scientific, Inc.). The transfected cells were collected after different periods of incubation and were subjected to the subsequent experiments.

### RT-qPCR

RNA isolation from the tissue specimens and cultured cells was performed using TRIzol® reagent (Invitrogen; Thermo Fisher Scientific, Inc.). The concentration of total RNA was determined on a NanoDrop 2000/2000C spectrophotometer (Invitrogen; Thermo Fisher Scientific, Inc.). To quantify miR-496 expression, reverse transcription was conducted to prepare first-strand complementary DNA (cDNA) using the miScript Reverse Transcription Kit (Qiagen GmbH, Hilden, Germany). Next, the miScript SYBR Green PCR Kit (Qiagen GmbH) was utilized for quantitative PCR on an ABI PRISM™ 7900 HT Sequence Detection System (Applied Biosystems, USA). The thermocycling conditions for qPCR were as follows: 95°C for 2 min, 95°C for 10 sec, 55°C for 30 sec and 72°C for 30 sec, for 40 cycles. The expression level of miR-496 was normalized to that of U6 small nuclear RNA.

For the measurement of *NNT-AS1* and *HMGB1* mRNA expression, cDNA was produced using the Prime-Script RT Reagent Kit (Takara Biotechnology Co., Ltd., Dalian, China). The synthesized cDNA was then subjected to PCR amplification with SYBR Premix Ex Taq (Takara Biotechnology Co., Ltd). The thermocycling conditions for qPCR were as follows: 5 min at 95°C, followed by 40 cycles of 95°C for 30 sec and 65°C for 45 sec. Glyceraldehyde-3-phosphate dehydrogenase (*GAPDH*) served as the normalization control for *NNT-AS1* and *HMGB1*. Relative gene expression was analyzed by the 2^−ΔΔCq^ method.

The primers for PCR were as follows: miR-496: forward, 5′-ACACTCCAGCTGGGAATGGAGGTTG TCCATGGTG-3′; reverse, 5′-CTCAACTGGTGTCGT GGAGTCGGCAATTCAGTTGAGGAGTACCG-3′; U6 forward, 5′-CGTTTTACTTCCTCATACAGCAC-3′; reverse, 5′-GCACCAAGAGACCTGTGACA-3′; NNT-AS1: forward, 5′- AGTTCCACCAAGTTTCTTCA-3′; reverse, 5′-AGGTTTTGCCAGCATAGAC-3′; HMGB1 forward, 5′-GCTCAGAGAGGTGGAAGACCA-3′; reverse, 5′-GGTGCATTGGGATCCTTGAA-3′; GAPDH forward, 5′-TGCACCACCAACTGCTTA-3′; reverse, 5′-GGATGCAGGGATGATGTTC-3′.

### CCK-8 assay

Transfected cells were collected 24 h after incubation, and a single-cell suspension was prepared with DMEM containing 10% of FBS to a final concentration of 2 × 10^4^ cells/ml. A total of 100 μl of the cell suspension was seeded in each well. Then, the cells were incubated at 37°C for 0, 24, 48, or 72 h, after which 100 μl of the CCK-8 solution (Sigma-Aldrich; Merck KGaA) was added into each well. After additional 2 h of incubation at 37°C and 5% CO_2_, absorbance was detected at an excitation wavelength of 450 nm on a microplate reader (Molecular Devices, Sunnyvale, CA, USA).

### Detection of apoptosis via flow-cytometric analysis

After 48 h of cultivation, transfected cells were detached using 0.25% trypsin without EDTA, washed with ice-cold PBS, and centrifuged at 4°C for 10 min. The proportion of apoptotic cells was determined with an Annexin V-Fluorescein Isothiocyanate (FITC) Apoptosis Detection Kit (Biolegend, San Diego, CA, USA). Briefly, cells were resuspended in 100 μl of binding buffer, and the suspension was then mixed with 5 μl of Annexin V-FITC and 5 μl of a propidium iodide solution, followed by 15 min incubation at room temperature in darkness. A FACScan flow cytometer (BD Biosciences, San Jose, CA, USA) was used to detect apoptotic cells.

### Transwell migration and invasion assays

Transwell chambers (8.0 μm pore size; BD Biosciences) precoated with Matrigel (BD Biosciences) were employed to evaluate the capacity of cells to invade. Transfected cells were harvested at 48 h post-transfection, and a cell suspension was prepared in FBS-free DMEM. The suspension concentration was adjusted to 2.5 × 10^5^ cells/ml. In total, 200 μl of the cell suspension was added into the upper compartments, while the lower compartments were covered with 800 μl of DMEM containing 20% of FBS. After 24 h of incubation, the noninvasive cells remaining on the upper surface were wiped off with a cotton swab. The cells that went through the pores and were located on the lower surface were fixed in 4% paraformaldehyde, stained with 0.1% crystal violet, and extensively washed. After drying, the invasive cells were imaged and subsequently counted under an inverted light microscope (Leica, Wetzlar, Germany). The average number of invasive cells was determined from five randomly selected visual fields and used to represent the invasive ability. The transwell migration assay was carried out in accordance with the same experimental procedures, but the chambers were not coated with Matrigel.

### Tumor xenograft assay

Female 6-week-old BALB/c nude mice were purchased from Shanghai Pharmaceutical Research Institute (Shanghai, China) and were subcutaneously injected with T24 cells harboring either siNNT-AS1 or siNC. Each group contained four nude mice. The animals were maintained under specific pathogen-free conditions (25°C, 50% humidity, 10-h light/14-h dark cycle) and libitum food/water access. Measurement of tumor volumes was started two weeks after the injection and carried out every 2 days. The volume of tumor xenografts was calculated using the following formula: tumor volume (mm^3^) = 0.5 × width^2^ (mm^2^) × length (mm). All the mice were euthanized via cervical dislocation at 4 weeks post injection, and the tumor xenografts were resected. After weighing, the tumor xenografts were stored in liquid nitrogen for further use.

### Subcellular fractionation

A PARIS Kit (Invitrogen; Thermo Fisher Scientific, Inc.) was utilized to separate the cytoplasmic and nuclear fractions of T24 and TCC-SUP cells. Total RNA was then isolated separately and subjected to RT-qPCR analysis for the determination of the intracellular distribution of *NNT-AS1*.

### RIP assay

A Magna RNA-binding Protein Immunoprecipitation Kit (Millipore, Billerica, MA, USA) was used to conduct a RIP assay to evaluate the binding interaction between *NNT-AS1* and miR-496 in bladder cancer cells. Cells were treated with RIP Lysis Buffer (Shanghai Haoran Biotechnology Co., Ltd., Shanghai, China). After 10 min of incubation followed by centrifugation at 4°C, the obtained cell extract was incubated with magnetic beads that were conjugated with either an anti–Argonaute 2 antibody (AGO2) or anti-IgG antibody (Millipore). Proteinase K was chosen to digest proteins prior to the isolation of immunoprecipitated RNA. The expression of *NNT-AS1* and miR-496 in the immunoprecipitated RNA was measured by RT-qPCR as described above.

### Bioinformatics analysis

A bioinformatic algorithm, starBase 3.0 (http://starbase.sysu.edu.cn/), was used to predict the miRNA interacting with *NNT-AS1*.

The putative target genes of miR-496 were predicted via three bioinformatic algorithms, including starBase 3.0, TargetScan (http://www.targetscan.org/), and miRDB (http://mirdb.org/).

### Luciferase reporter assay

The fragment of *NNT-AS1* containing either the wild-type (wt) or mutant (mut) miR-496–binding sequence (one of two versions) was chemically synthesized by Shanghai GenePharma Technology and inserted into the pmirGLO luciferase reporter plasmid, resulting in the plasmids wt-NNT-AS1 and mut-NNT-AS1, respectively. The reporter plasmids wt-HMGB1 and mut-HMGB1 were constructed in the same way. For the reporter assay, the miR-496 mimics or miR-NC plus wt or mut reporter plasmid was introduced into cells using Lipofectamine® 2000. The firefly luciferase activity was detected via a Dual-Luciferase® Reporter Assay System (Promega, Madison, WI, USA) and was normalized to *Renilla* luciferase activity.

### Western blot analysis

Total protein was extracted from tissues or cells using RIP Lysis Buffer, after which protein concentration was quantified with the BCA Protein Assay Kit (Beyotime Institute of Biotechnology, Shanghai, China). Equal amounts of protein were separated by gel electrophoresis using an SDS–polyacrylamide 10% gel and transferred to polyvinylidene difluoride (PVDF) membranes. The membranes were then blocked with 5% fat-free milk in Tris-buffered saline containing 0.1% Tween 20 (TBST) at room temperature for 2 h, followed by overnight incubation at 4°C with a primary antibody against either HMGB1 (ab79823; 1:1000 dilution; Abcam, Cambridge, MA, USA) or GAPDH (ab181602; 1:1000 dilution; Abcam). After three rinses with TBST, a horseradish peroxidase–conjugated goat anti-rabbit IgG secondary antibody (ab205718; 1:5000 dilution; Abcam) was added to the membrane and incubated for another 2 h at room temperature. The protein signals were detected using Pierce™ ECL Western Blotting Substrate (Pierce Biotechnology, Inc., Rockford, IL, USA). GAPDH served as the loading control.

### Statistical analysis

Each assay was repeated at least three times. All the data are presented as the mean ± standard deviation (SD) and were analyzed with SPSS 21.0 software (IBM Corp., Armonk, NY, USA). The correlations between *NNT-AS1* levels and clinical characteristics of the patients with bladder cancer were determined using the chi-square (χ^2^) test. Spearman's correlation analysis was employed to test the expression correlation between *NNT-AS1* and *miR-496* in bladder cancer tissues. Student’s *t* test was carried out for evaluating the differences between two groups. Comparisons among multiple groups were conducted via one-way analysis of variance followed by Tukey’s test. The association of *NNT-AS1* with the overall survival of patients with bladder cancer was tested by the Kaplan–Meier method and log rank test. Data with a P value < 0.05 were considered statistically significant.
